# Integrating proteomics to map immune landscape and protein biomarkers for risk stratification in childhood Langerhans cell histiocytosis

**DOI:** 10.3389/fimmu.2026.1909960

**Published:** 2026-10-21

**Authors:** Jinqi Liu, Huizhuang Shan, Jiaguo Yuan, Weina Zhang, Qingqing Zheng, Haiyan Liu, Pengfei Wang, Huiling Lin, Caiyun Kuang, Meifen Huang, Kaoxian Guo, Jiayi Wang, Hua Jiang, Xinhua Xiao

**Affiliations:** 1 Department of Hematology and Oncology, Guangzhou Women and Children’s Medical Center, Guangzhou Medical University, Guangzhou, China; 2 Department of Clinical Laboratory Medicine, Guangdong Provincial People’s Hospital (Guangdong Academy of Medical Sciences), Southern Medical University, Guangzhou, Guangdong, China; 3 Department of Ophthalmology, The Third Affiliated Hospital of Chongqing Medical University, Chongqing, China

**Keywords:** biomarkers, immune landscape, Langerhans cell histiocytosis, machine learning, proteomics, risk stratification

## Abstract

**Background:**

Langerhans cell histiocytosis (LCH) is a rare pediatric disease with marked clinical heterogeneity. Current risk stratification relies on the extent of organ involvement and risk-organ status, yet reliable protein biomarkers for distinguishing LCH subtypes are lacking.

**Methods:**

We performed proteomic analysis on 56 formalin‐fixed paraffin‐embedded (FFPE) pediatric LCH tissue samples and 10 normal skin controls, quantifying 6, 914 proteins. Machine-learning approaches were integrated with weighted correlation network analysis for exploratory identification of candidate protein biomarkers and internal classification analysis. Candidate markers were validated by immunohistochemistry. The CIBERSORT deconvolution algorithm was employed to estimate the immune cell infiltration.

**Results:**

Proteomic profiling clearly distinguished LCH from controls. Functional protein modules and candidate protein biomarkers were identified across different LCH subtypes. Significant differences in immune cell infiltration were also observed, revealing enhanced innate immune activation and cytotoxic T cell responses in multisystem LCH compared with single-system LCH, as well as in LCH with risk-organ involvement versus those without. In exploratory internal classification analyses, the SVM model yielded an accuracy, AUC, and F1 score of 1.000 (95% CI: 1.000–1.000) for distinguishing multisystem from single-system LCH. For risk-organ involvement, the KNN model yielded an accuracy of 0.800 (95% CI: 0.500–1.000) and an AUC of 0.880 (95% CI: 0.600–1.000).

**Conclusion:**

To our knowledge, this is the first large-scale FFPE-based proteomic study in pediatric LCH to reveal subtype and risk specific protein signatures and associated immune cell profiles changes. These findings provide novel biological insights into LCH heterogeneity and identify candidate protein biomarkers associated with disease extent and risk-organ involvement. Further validation in larger independent cohorts is required before their predictive or clinical utility can be established.

## 1 Introduction

Langerhans cell histiocytosis (LCH) is a rare disease characterized by pathological infiltration of CD1a^+^/CD207^+^ cells. It exhibits an overall annual incidence of approximately 5 cases per million that predominantly affects children, with an incidence of 4.6 cases per million in individuals under 15 years old and 1 case per million in adults (1). LCH exhibits diverse clinical manifestations and can involve multiple organ systems. The disease is classified into single-system LCH (SS-LCH) and multisystem LCH (MS-LCH) based on the number of involved organs and sites (2). Common sites of LCH involvement include the skin, bones, lungs, liver, and pituitary gland. Among these, the liver, spleen, and bone marrow are defined as high-risk organs (3). Multisystem LCH can be further categorized into high-risk organ involvement [LCH-RO (+)] and no high-risk organ involvement [LCH-RO (–)] based on whether these organs are affected (4). Notably, MS-LCH patients, especially those with high-risk organ involvement, exhibit significantly higher disease-related mortality compared to single-system LCH patients (5, 6). The clinical presentation of LCH is highly heterogeneous, ranging from isolated self-limiting lesions to progressive multi-organ involvement with significant morbidity and mortality. Sequelae may include substantial damage to the endocrine, auditory, skeletal, neurocognitive, pulmonary, and hepatic systems (3, 7).

Several prognostic biomarkers have been identified in LCH. At the genetic level, the BRAF gene is currently the only gene with recurrently validated somatic mutations in multiple patients, with the BRAF V600E point mutation detected in approximately 50%-65% of LCH lesions (8, 9). The presence of the BRAF V600E mutation is significantly associated with multisystem LCH involving high-risk organs, disease progression, or recurrence, making it an important genetic biomarker (10–12). Additionally, cytokine levels such as IL-6, IL-10, IL-18, TNF-α, and soluble CD25 (sCD25) have been established as important serological biomarkers for assessing systemic inflammation, disease activity, and prognosis (13–16).

The striking histological uniformity of LCH lesions makes it impossible for pathologists to determine whether a biopsy specimen comes from a patient with single-system or multisystem disease, or to assess whether the case represents a moderate-risk or high-risk condition (17). Consequently, the development of additional biomarkers for risk stratification in LCH is both necessary and clinically significant.

Through proteomic analysis, this study systematically characterized the molecular features of LCH across different clinical subtypes, including single-system and multisystem disease, as well as risk-organ involvement status. We identified multiple differentially expressed functional modules and candidate protein biomarkers. BICDL2, CD22, CLDN4, DSC3, ELP1, and NOSIP were associated with differences between single-system and multisystem LCH, whereas DOK3, RPL18, and IDI1 were associated with risk-organ involvement. Exploratory machine-learning analyses were further performed to assess whether these candidate proteins contained classification information related to disease extent and risk-organ status. These findings identify candidate protein biomarkers associated with LCH clinical heterogeneity and provide a molecular basis for future validation in independent cohort.

## 2 Materials and methods

### 2.1 Human sample preparation

Formalin-fixed paraffin-embedded (FFPE) tissue samples used in this study were obtained from surgical resections of pediatric LCH patients at the Guangzhou Women and Children’s Medical Center affiliated with Guangzhou Medical University. All cases were confirmed by pathological diagnosis at the same institution. Diagnosis strictly followed the 2009 International Histiocyte Society guidelines for evaluation and treatment of LCH (18). The study was conducted in accordance with the ethical principles of the Declaration of Helsinki. Clinical data were anonymized to protect patient privacy, and the study protocol was approved by the Institutional Review Board of Guangzhou Women and Children’s Medical Center (Ethics Approval No.: [2025]008A01). The sample groups were as follows: Skin control (n = 10), LCH (n = 56), SS-LCH (n = 25), MS-LCH (n = 31), LCH-RO (−) (n = 17), and LCH-RO (+) (n = 18) (**Supplementary Table 1**).

### 2.2 Micro-proteomic sample preparation

FFPE sections of 5 μm thickness were prepared, with 1–2 sections per case. After dewaxing, BT lysis buffer, ABC solution, and PMSF were sequentially added to cover the tissue, followed by non-contact ultrasonication for 10 min at 4 °C and brief centrifugation to collect the solution. The samples were then heated at 90 °C with shaking at 1500 rpm for 15 min, with brief centrifugation every 5 min to prevent drying, and rapidly cooled to room temperature. Subsequently, DTT was added and incubated at 55 °C for 30 min, followed by IAA incubation at room temperature in the dark for 30 min. ABC solution was added to adjust the BT concentration to <0.5%. Finally, Trypsin was added for enzymatic digestion at 37 °C with shaking at 1500 rpm. The digested products were desalted and peptide concentration was quantified using Nanodrop.

### 2.3 LC-MS/MS high-resolution mass spectrometry

Prior to MS injection, each sample was mixed with iRT standard (Biognosys, ThermoFisher) at a volume ratio of iRT:sample = 1:20. The iRT standard consists of 11 synthetic peptides not found in nature, optimized for stability, sensitivity, and retention time, and does not interfere with the sample. Each fraction was separated using a nanoElute liquid chromatography system (Bruker) with mobile phase A (0.1% formic acid in water) and mobile phase B (0.1% formic acid in acetonitrile). The gradient elution conditions were: 0 min, 6% B; 7.5 min, 28% B; 9 min, 80% B; 10 min, 80% B. Peptides were injected into a timsTOF Pro mass spectrometer (Bruker) with the following settings: capillary voltage 1600 V, MS1 and MS2 scan range 300–1500 m/z, and ion mobility window (1/K0) 0.7–1.3 Vs/cm². LC-MS/MS analysis was performed by Shanghai OE Biotech Co., Ltd.

### 2.4 Protein identification, quantification, and analysis

All MS data were processed using PASER software, followed by database search and DIA-based protein quantification. The search database was uniprot-Homo sapiens-9606-irt-2024.2.1.fasta. MS search parameters included precursor mass tolerance 15 ppm, protein FDR threshold 0.01, fixed modification Carbamidomethyl (C), variable modifications Oxidation (M) and Acetyl (N-term), and maximum missed cleavages = 1. Raw data from database search were filtered to retain proteins with Unique Peptides ≥1, valid values in ≥2 samples, and at least one group with ≥50% valid value proportion. Missing values in groups with ≥50% valid proportion were imputed with the group mean; remaining missing values were filled with half the minimum value of the matrix. After median normalization and log_2_ transformation, reliable proteins were obtained.

### 2.5 Differential protein and functional enrichment analysis

Differential analysis was performed using the limma R package (version 3.66.0) (19). Differential proteins were defined as those with P < 0.05 and |log_2_FC| > 0.58; proteins with P < 0.05 and log_2_FC > 0.58 were considered significantly upregulated, while those with P < 0.05 and log_2_FC < -0.58 were considered significantly downregulated. Gene Ontology (GO) pathway enrichment analysis of DEGs was performed based on the hypergeometric distribution using OECloud tools (https://cloud.oebiotech.cn) (20).

### 2.6 WGCNA analysis

This study used the WGCNA R package (version 1.72-5) to identify key protein co-expression modules in each sample group. The workflow included: hierarchical clustering of samples and removal of outliers; determination of the optimal soft-threshold (β) using the “pickSoftThreshold” function to convert the correlation matrix into an adjacency matrix, which was then transformed into a topological overlap matrix (TOM). Based on the TOM matrix, average-linkage hierarchical clustering was used to construct a dendrogram, and dynamic tree-cutting (minModuleSize = 30) was applied to identify co-expression modules. Structurally similar modules were merged. Finally, Spearman correlation coefficients were used to evaluate the association between module eigengenes and clinical traits.

Functional annotation and enrichment scoring for each module were computed based on the hypergeometric distribution using OECloud tools (https://cloud.oebiotech.cn) (20). Network construction and visualization were performed using magrittr (2.0.3), igraph (1.3.1), ggraph (2.1.0), and purrr (1.0.2). Single-sample GSEA (ssGSEA) was carried out using GSVA (1.46.0) and GSEABase (1.60.0), and visualization was performed using ggplot2 (3.5.1), ggpubr (0.4.0), and ggthemes (5.0.0). Representative pathways were selected based on ascending P-values from the hypergeometric test.

### 2.7 Immune cell infiltration analysis

To estimate the relative proportions of immune cells in each sample, the CIBERSORT deconvolution algorithm was employed. According to the CIBERSORT website (http://cibersort.stanford.edu/), the LM22 signature matrix contains gene expression signatures of 22 immune cell types. We used the CIBERSORT R package (version 0.1.0) with the LM22 signature to quantify the immune cell composition of each sample. UniProt protein identifiers were first mapped to approved official human gene symbols. Duplicate protein entries mapped to the same gene were then summarized, after which the resulting gene-level protein abundance matrix was intersected with the LM22 signature genes before CIBERSORT analysis. Only samples with CIBERSORT *P* < 0.05 were retained for further analysis. The estimated immune cell proportions were normalized to sum to 1 for each sample to enable comparison across immune cell types and datasets. Immune cell composition and correlation results were visualized using the R packages “corrplot” (version 0.92), “vioplot” (version 0.4.0), and “ggplot2” (version 3.5.1). Additionally, non-parametric Spearman correlation analysis was used to evaluate the relationship between immune infiltrating cells and diagnostic biomarker proteins.

### 2.8 Key protein screening via machine learning

Proteins that were both significantly differentially expressed and belonged to the most relevant WGCNA modules in each group were selected as the input matrix for machine learning. Three established machine learning algorithms-LASSO, SVM-RFE, and Random Forest (RF)-were applied to further screen proteins within each group. To ensure reproducibility, a fixed random seed (123) was set for all analyses. First, the protein matrix was input into LASSO regression using the R package “glmnet” (version 4.1-8) with family = “binomial” and 10-fold cross-validation (CV). The optimal λ value was selected by “lambda.1min”. The coefficient path plot and binomial deviance versus log(λ) curve were generated. Next, SVM-RFE was performed using the R package “e1071” (version 1.7-14) to eliminate irrelevant features. All identified proteins were included in the SVM model, and the SVM-RFE results were visualized. In the 5-fold CV, the point marked with a red circle represented the highest classification accuracy, and the corresponding protein set was considered the most accurate diagnostic marker, showing the lowest 5×CV error rate and highest 5×CV accuracy. Finally, the Random Forest R package (version 4.7-1.1) was used for classification based on protein importance. A random forest model with 500 trees was built in the discovery cohort, and the optimal number of trees was determined by cross-validation error. The top 50 important proteins were selected. The intersection of results from the three algorithms was taken. Candidate proteins were determined as the target diagnostic proteins, and a Venn diagram was drawn using the R package “ggvenn” (version 0.1.10).

### 2.9 Predictive model construction

Machine-learning models were developed using the previously identified core protein biomarkers to distinguish multisystem from single-system LCH and to identify patients with risk-organ involvement. Candidate proteins were selected from the complete discovery cohort and used as a fixed feature panel for subsequent model development and validation. All analyses were performed in R software (version 4.6.0). Stratified random sampling with a fixed random seed was used to divide the data into training and internal evaluation set at a 7:3 ratio while preserving class proportions. Multisystem LCH and LCH-RO (+) were defined as the positive classes for the corresponding tasks. Missing-value imputation, removal of low-variance features, centering, and scaling were performed in the training set, and the resulting parameters were subsequently applied to the validation set. Seven algorithms were compared: logistic regression, support vector machine (SVM), random forest (RF), XGBoost, naïve Bayes (NB), k-nearest neighbors (KNN), and multilayer perceptron (MLP). Logistic regression, SVM, and NB were implemented using the caret package (version 6.0-94), with a radial basis function kernel used for SVM. RF was constructed using the randomForest package (version 4.7-1.1) with 500 trees; XGBoost was implemented using the xgboost package; KNN was constructed using the class package (version 7.3-22); and MLP was developed using the nnet package (version 7.3-19) with five hidden neurons and a weight-decay parameter of 0.1. Hyperparameters were optimized using stratified five-fold cross-validation within the training set.

Model stability based on the fixed feature panel was further assessed using repeated nested cross-validation. The outer loop comprised stratified five-fold cross-validation repeated 20 times to generate out-of-fold predictions, whereas the inner loop used stratified five-fold cross-validation for hyperparameter selection and model training. Performance was evaluated separately in the training and internal evaluation set using accuracy, the area under the receiver operating characteristic curve (AUC), and the F1 score, with 95% confidence intervals estimated from 2, 000 stratified bootstrap resamples. Representative models were selected according to their overall performance in the internal evaluation set and the consistency between the training and validation results. ROC curves, calibration curves, and decision curve analyses were generated using out-of-sample predicted probabilities. Calibration was assessed using the Brier score, calibration intercept, and calibration slope, whereas decision curve analysis compared the net benefits of the model-based, treat-all, and treat-none strategies across a range of threshold probabilities.

### 2.10 Immunohistochemistry and quantification

FFPE tissue sections were cut into single thin slices and subjected to dewaxing, antigen retrieval, endogenous peroxidase blocking, and serum blocking. Primary antibodies at appropriate dilutions were added and incubated overnight at 4 °C in a humidified chamber, followed by incubation with species-matched secondary antibodies at room temperature for 50 min. Staining was developed with DAB, counterstained with hematoxylin, dehydrated, and mounted. The antibodies used for immunohistochemistry were BICDL2 (#22770-1-AP), DOK3 (#15749-1-AP), and DSC3 (#83386-5-RR), all purchased from Proteintech. The H-score, a histological scoring method for semi-quantitative assessment of immunohistochemistry, integrates both the proportion of positive cells and staining intensity. The formula is: H-score = (percentage of weakly stained cells × 1) + (percentage of moderately stained cells × 2) + (percentage of strongly stained cells × 3). H-scores for BICDL2, DOK3, and DSC3 were calculated using the Aipathwell digital pathology image analysis software (Servicebio^®^) (21).

### 2.11 Statistical analysis

Statistical analysis was performed using R (version 4.4.0). Differential expression analysis was conducted using the limma package (version 3.66.0), with proteins meeting the criteria of *P*< 0.05 and |log_2_FC| > 0.58 considered differentially expressed. Immune infiltration was estimated via the CIBERSORT package (version 0.1.0), and associations between protein markers and immune cells were assessed using Spearman correlation. Immunohistochemistry results were quantified by H-score and compared using the Wilcoxon test. *P* < 0.05 was considered statistically significant.

## 3 Results

### 3.1 Pediatric patients with Langerhans cell histiocytosis

To elucidate the proteomic characteristics of LCH patients and identify biological protein markers for risk stratification, we collected 56 paraffin-embedded tissue (FFPE) samples from patients with confirmed LCH diagnoses, along with 10 normal skin tissue samples as controls, for in-depth proteomic analysis. By integrating proteomic differences among SS-LCH, MS-LCH, LCH-RO (+), and LCH-RO (–) groups (**Supplementary Table 1**), we constructed machine learning models to aid in diagnosis and prediction (**Figure 1A**). LC-MS/MS identified 6914 proteins in the LCH samples. Principal component analysis (PCA) revealed a clear separation between LCH samples and control skin samples in overall protein expression profiles (**Figure 2B**), indicating significant heterogeneity in protein expression between the two groups. Compared to controls, LCH samples exhibited upregulation of 1630 proteins and downregulation of 1372 proteins (**Figure 2C**; **Supplementary Table 2**). GO functional enrichment analysis showed that upregulated proteins were primarily involved in processes such as cytosol, nucleoplasm, mitochondrial large ribosomal subunit, RNA binding, and mitochondrial translation, which are associated with intracellular protein synthesis activation and mitochondrial energy production. In contrast, downregulated proteins were enriched in pathways related to skin epidermal barrier structure and extracellular matrix function, including collagen-containing extracellular matrix, keratinization, intermediate filament organization, cornified envelope, and structural constituent of skin epidermis (**Figure 2D**).

**Figure 1:**
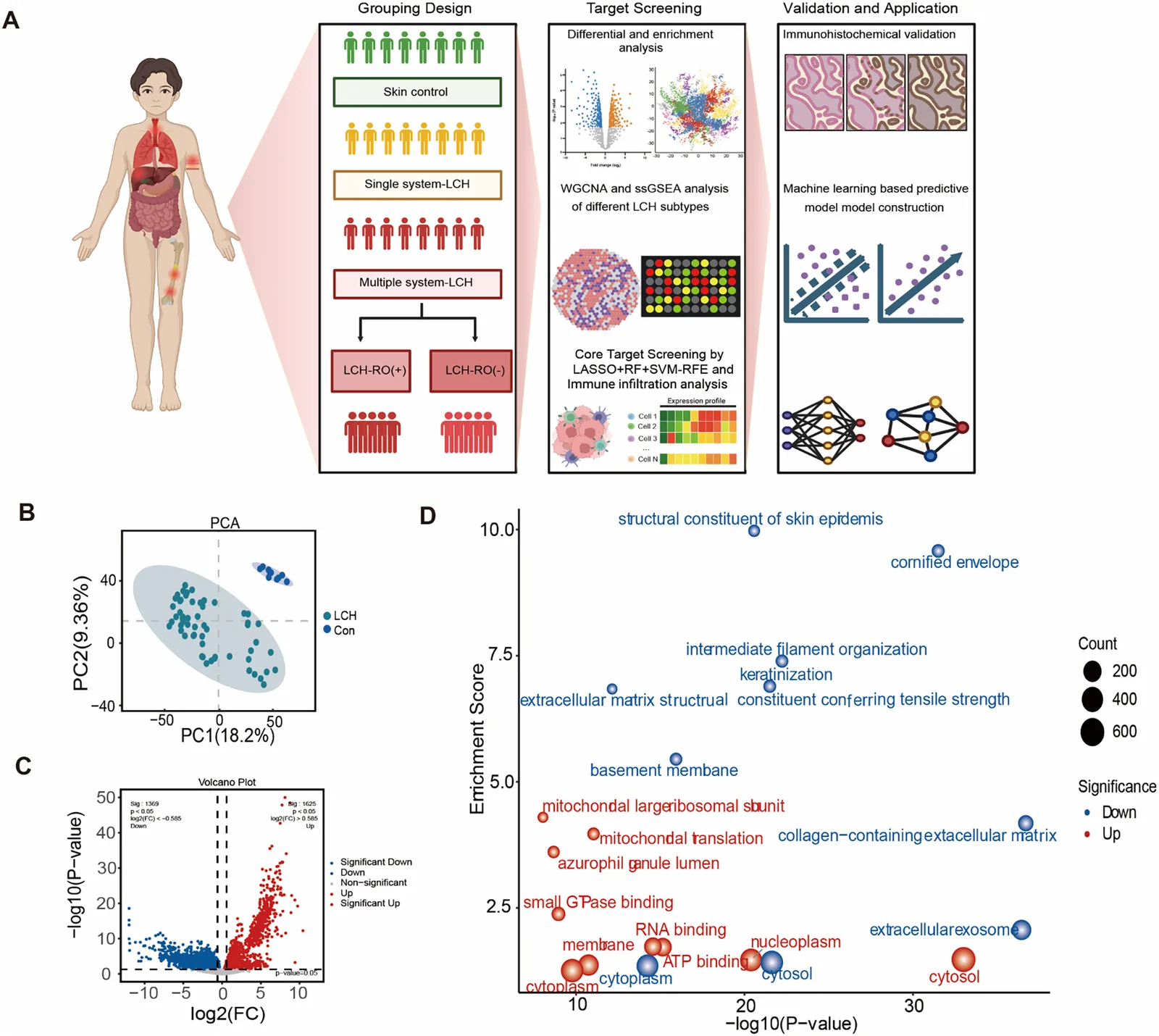
Overview of the study design and pathway characteristics in Langerhans cell histiocytosis (LCH). **(A)** Workflow illustrating the integration of LCH clinical classification, risk stratification, and proteomic data. **(B)** Principal component analysis (PCA) showing the distribution of LCH samples and control skin samples. Each point represents one sample: blue points indicate LCH samples, and light green points indicate skin samples. The percentage values represent the variance explained by each principal component. **(C)** Volcano plot displaying differentially expressed proteins between MS-LCH and SS-LCH. Each point represents a protein: red points indicate significantly upregulated proteins, and blue points indicate significantly downregulated proteins. Significance thresholds were defined as: upregulated in MS-LCH (*P* < 0.05 and log_2_FC > 0.58, i.e., FC > 1.5), downregulated in MS-LCH (*P* < 0.05 and log_2_FC < −0.58), and nonsignificant otherwise. **(D)** Dot plot showing enriched pathways based on significantly upregulated/downregulated proteins. Red dots represent pathways significantly enriched for upregulated proteins in LCH compared to skin controls (*P* < 0.05), while blue dots represent pathways significantly enriched for downregulated proteins (*P* < 0.05).

**Figure 2:**
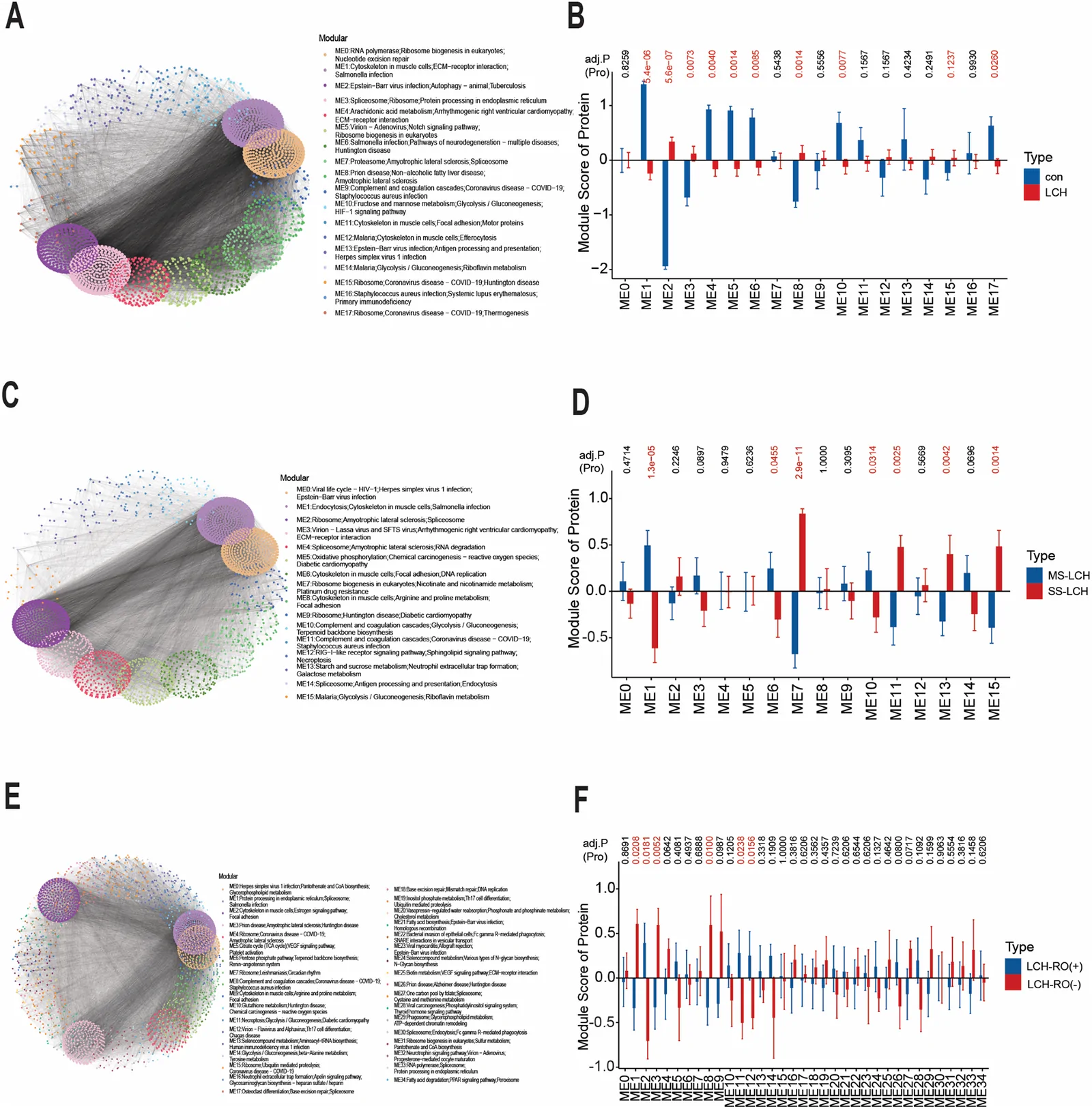
Proteomic landscape and clinical relevance of functional protein modules in LCH. **(A)** Eighteen functional protein modules identified by WGCNA in the comparison between LCH and control skin samples. Each network node represents a protein, colored according to its functional module. **(B)** Normalized enrichment scores (NES) of protein modules in LCH versus control skin samples, showing differences in module activity between the two groups. **(C)** Sixteen functional protein modules identified by WGCNA in the comparison between MS-LCH and SS-LCH samples. **(D)** NES of protein modules in MS-LCH versus SS-LCH, illustrating differences in module activity. **(E)** Thirty-five functional protein modules identified by WGCNA in the comparison between LCH-RO (+) and LCH-RO (−) samples. **(F)** NES of protein modules in LCH-RO (+) versus LCH-RO (−), showing differences in module activity between the two groups. *P*-values were calculated using the Wilcoxon test, and adjusted *P*-values were computed using the Benjamini-Hochberg (BH) method. Red indicates adjusted *P* < 0.05.

To evaluate the potential influence of anatomical tissue origin on the proteomic findings, we further examined the global proteomic profiles of LCH lesions according to biopsy site. PCA revealed partial separation between skin- and skeleton-derived LCH lesions, particularly along PC1, although substantial overlap remained between the groups. PC1 and PC2 explained 17.3% and 6.7% of the total variance, respectively (**Supplementary Figure S1A**). PERMANOVA further demonstrated a significant association between biopsy site and overall proteomic composition (R² = 0.114, F = 3.416, *P* = 0.001), indicating that anatomical origin accounted for approximately 11.4% of the total proteomic variation (**Supplementary Figure S1A**). To further assess whether the disease-versus-control differences were primarily driven by tissue origin, we performed a sensitivity analysis restricted to skin-derived LCH lesions and normal skin controls. Protein-level effect sizes obtained from this tissue-matched comparison showed a strong positive correlation with those obtained from the primary analysis including all LCH lesions (Spearman ρ = 0.882, *P* < 2 × 10^-6^, **Supplementary Figure S1B**). These results indicate that, although biopsy site contributed to overall proteomic heterogeneity, the major proteomic alterations observed in the primary disease-versus-control comparison were largely preserved after matching tissue origin.

To assess the potential influence of biopsy site on proteomic differences across LCH clinical subtypes, we performed sensitivity analyses stratified by anatomical site. Of the 56 LCH samples with biopsy-site information, 36 were from skeletal lesions, 19 from skin lesions, and 1 from another anatomical site. We therefore repeated the differential protein analyses for MS-LCH versus SS-LCH and LCH-RO (+) versus LCH-RO (−) separately within the skeleton-derived and skin-derived samples, and compared the resulting protein-level effect sizes with those obtained from the full cohort. The results of the differential analyses between the groups are provided in **Supplementary Table 3**. For the MS-LCH versus SS-LCH comparison, the log2 fold changes from the skeleton- and skin-stratified analyses were significantly correlated with those from the overall analysis (Spearman ρ = 0.787 and 0.737, respectively; both *P* < 0.001). For the LCH-RO (+) versus LCH-RO (−) comparison, the corresponding correlation coefficients were 0.541 and 0.814, respectively (both *P* < 0.001) (**Supplementary Figure S2**).

Further differential analysis of proteomic results between MS-LCH and SS-LCH patient samples revealed 713 upregulated and 887 downregulated proteins (**Supplementary Figure S3A**; **Supplementary Table 2**). GO enrichment analysis indicated that pathways such as structural constituent of skin epidermis, keratinization, extracellular exosome, collagen-containing extracellular matrix, and intermediate filament organization were activated in MS-LCH (**Supplementary Figure S3B**). Additionally, differential analysis between LCH-RO (+) and LCH-RO (–) samples identified 780 upregulated and 913 downregulated proteins (**Supplementary Figure S3C**; **Supplementary Table 2**). GO enrichment analysis showed that structural constituent of skin epidermis, keratinization, collagen-containing extracellular matrix, and intermediate filament organization were similarly activated in LCH-RO (+) patients (**Supplementary Figure S3D**). These findings suggest that LCH samples with different systemic involvement statuses and risk levels consistently exhibit proteomic alterations characterized by the activation of epidermal development and keratin-related pathways. This indicates abnormalities in epithelial keratinization and barrier formation-related biological processes, which may play a significant role in LCH disease progression.

### 3.2 Differences in functional protein modules across LCH subtypes

We performed weighted correlation network analysis (WGCNA) (22) on proteomic data obtained from LCH and normal skin control samples. To ensure the network satisfied scale-free topology criteria, a soft threshold power of 4 was selected (**Supplementary Figure S4A**). A total of 18 functional modules were identified, each associated with distinct biological processes and molecular pathways. These modules were significantly enriched in biological processes including viral or bacterial infection (e.g., ME2, ME5, ME6, ME9, ME13, ME15, ME17), immune response (e.g., ME13, ME9), metabolism (e.g., ME4, ME10, ME14), cytoskeletal dynamics (e.g., ME1, ME11, ME12), and protein synthesis (e.g., ME3, ME15, ME17) (**Figure 2A**). Notably, modules ME2, ME3, and ME8 were significantly enriched in LCH samples and exhibited positive single-sample gene set enrichment analysis (ssGSEA) scores, suggesting their activation in LCH, while six modules, such as ME1, ME4, and ME5, were downregulated (**Figure 2B**). The module-trait correlation heatmap revealed that module ME1 showed a significant negative correlation with LCH (r = -0.53), whereas module ME3 showed a significant positive correlation with LCH (r = 0.62) (**Supplementary Figure S4B**).

We further constructed weighted co-expression networks based on protein expression matrices for MS-LCH versus SS-LCH, and for LCH-RO (+) versus LCH-RO (–) samples. The soft threshold powers were set to 3 for MS-LCH versus SS-LCH and to 4 for LCH-RO (+) versus LCH-RO (–) to satisfy scale-free topology criteria (**Supplementary Figures S4C, E**). In the comparison of MS-LCH versus SS-LCH, 16 functional modules were identified. These modules were significantly enriched in key biological processes such as viral infection (e.g., ME0, ME3, ME11), immune response (e.g., ME10, ME12), metabolism (e.g., ME5, ME10, ME13, ME15), and cytoskeletal dynamics (e.g., ME1, ME6, ME8) (**Figure 2C**). Modules ME1, ME6, and ME10 were significantly upregulated in multisystem LCH, whereas modules ME7, ME11, ME13, and ME15 were significantly downregulated (**Figure 2D**). The module-trait correlation heatmap showed that in the comparison of MS-LCH versus SS-LCH, module ME1 exhibited the strongest positive correlation with MS-LCH (r = 0.86), and module ME7 showed the strongest negative correlation (r = -0.86) (**Supplementary Figure S4D**).

Protein molecular differences between LCH-RO (+) and LCH-RO (–) samples were clustered into 35 functional modules. These modules were significantly enriched in key pathways, including viral infection (e.g., ME12, ME13, ME21, ME23), immune inflammation (e.g., ME8, ME16, ME19), metabolism (e.g., ME5, ME10, ME11, ME14), and cytoskeletal regulation (e.g., ME2, ME9) (**Figure 2E**). Modules ME1, ME3, and ME8 were significantly upregulated in LCH-RO (–), while modules ME2, ME11, and ME12 were significantly upregulated in LCH-RO (+) (**Figure 2F**). The module-trait correlation heatmap revealed that in the comparison of LCH-RO (+) versus LCH-RO (–), modules ME22 and ME4 showed the strongest positive correlation with LCH-RO (+) (r = 0.48), whereas module ME12 showed the strongest negative correlation with LCH-RO (+) (r = -0.51) (**Supplementary Figure S4F**).

### 3.3 Machine learning-based identification of core differentially expressed proteins in multisystem LCH and single-system LCH

In the comparative analysis between MS-LCH and SS-LCH, we first intersected differentially expressed proteins with module proteins from WGCNA most strongly associated with disease subtypes, yielding 727 candidate feature proteins (**Figure 3A**). This protein set was designated as the core input dataset for subsequent machine learning analysis. We then systematically screened candidate protein biomarkers using three distinct algorithms: LASSO regression, random forest (RF), and support vector machine recursive feature elimination (SVM-RFE). All three methods convergently identified seven stable protein biomarkers that showed consistent importance across algorithms (**Figure 3B**). Specifically, LASSO regression (with λ = 0.004419677) selected 23 proteins with non-zero regression coefficients; SVM-RFE, under the condition of minimal 5-fold cross-validation error and highest cross-validation accuracy, identified 662 feature genes (**Supplementary Figures S5A–D**; **Figure 3B**); and RF, applied to the aforementioned 727 proteins, ranked the top 50 key proteins by importance (**Supplementary Figure S5E**). All seven protein biomarkers exhibited significant differential expression between MS-LCH and SS-LCH samples: BICDL2, CD22, CLDN4, DSC3, ELP1, and NOSIP were markedly upregulated in MS-LCH, while NAGLU was significantly downregulated (**Figure 3C**). To further validate their reliability, we randomly selected BICDL2 and DSC3 for immunohistochemical staining in MS-LCH and SS-LCH tissue samples. The results confirmed high expression of both BICDL2 and DSC3 in MS-LCH tissues and low expression in SS-LCH tissues (**Figures 3D, F**). H-Score semi-quantitative analysis further substantiated this expression pattern (**Figures 3E, G**).

**Figure 3:**
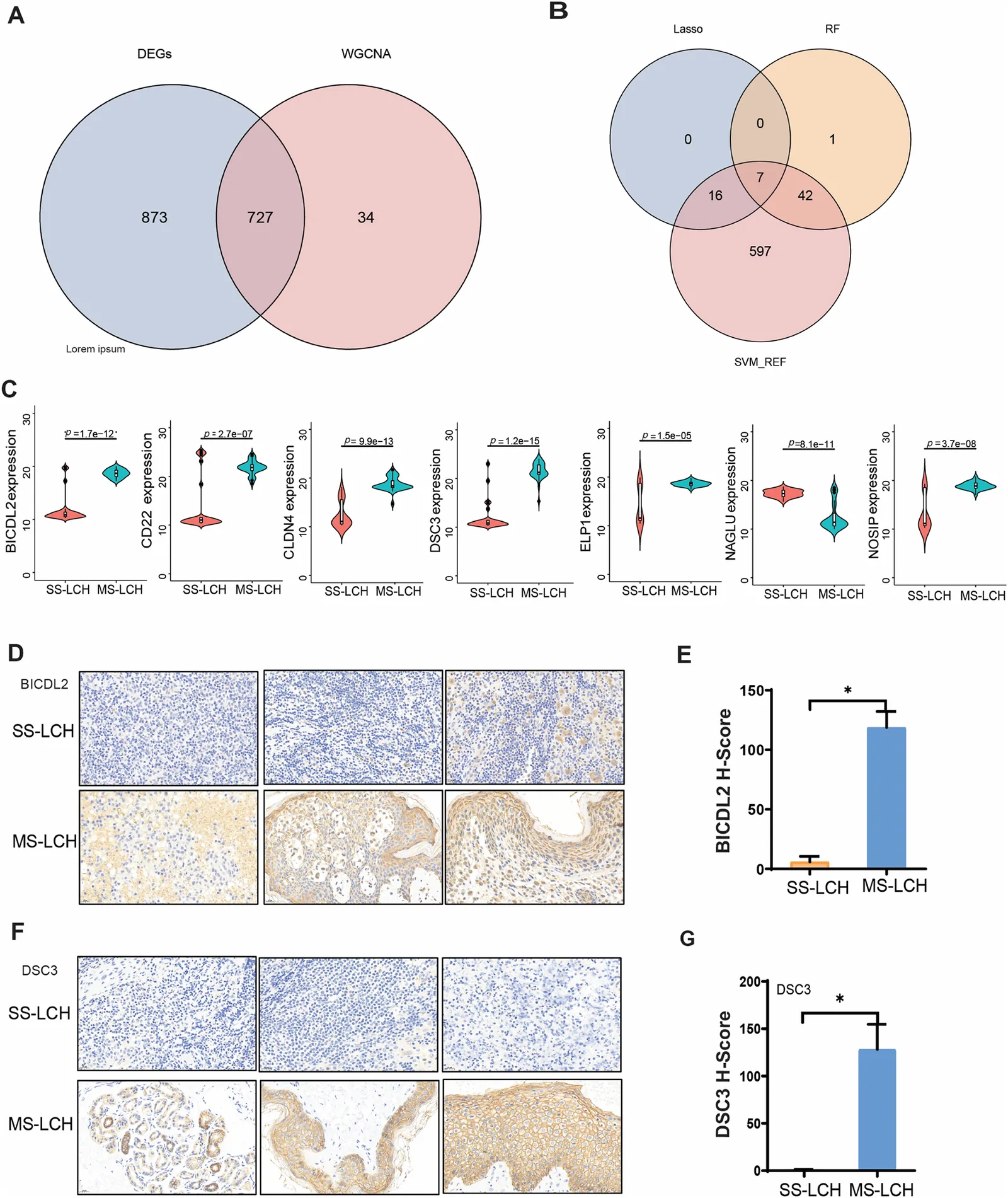
Screening and validation of protein biomarkers for multisystem-LCH versus single-system-LCH. **(A)** Candidate feature proteins were selected by intersecting differentially expressed proteins with key module proteins from WGCNA. **(B)** Feature selection was performed using LASSO regression, random forest, and support vector machine recursive feature elimination (SVM-RFE). A Venn diagram shows the seven core protein biomarkers commonly identified by all three methods. **(C)** Violin plots display the expression levels of the seven core proteins-BICDL2, CD22, CLDN4, DSC3, ELP1, and NOSIP-in SS-LCH and MS-LCH tissues. **(D)** Immunohistochemical staining images of BICDL2 and DSC3 in MS-LCH and SS-LCH tissues. **(E, G)** H-Score semi-quantitative analysis of BICDL2 and DSC3 expression. *P* < 0.05 indicates statistical significance. * P < 0.05, ** P < 0.01, and *** P < 0.001.

### 3.4 Machine learning-based identification of core differentially expressed proteins in LCH-RO (+) and LCH-RO (–)

To identify core differentially expressed proteins between LCH-RO (+) and LCH-RO (–) samples, we first integrated differentially expressed proteins with WGCNA module proteins most closely associated with risk stratification, resulting in 602 candidate features for machine learning input (**Figure 4A**). Feature selection was subsequently performed using three algorithms: LASSO regression, random forest (RF), and support vector machine recursive feature elimination (SVM-RFE). All three methods convergently identified three robust candidate protein biomarkers (**Figure 4B**), indicating high consistency of these core features across different machine learning approaches. Specifically, LASSO regression (with λ = 0.01571872) selected 16 proteins with non-zero regression coefficients; SVM-RFE, under the condition of minimal 5-fold cross-validation error and highest cross-validation accuracy, identified 91 feature proteins (**Supplementary Figures S5F–I**; **Figure 4B**); and RF, applied to the aforementioned 727 proteins, ranked and confirmed the top 50 key proteins by importance (**Supplementary Figure S5J**).

**Figure 4:**
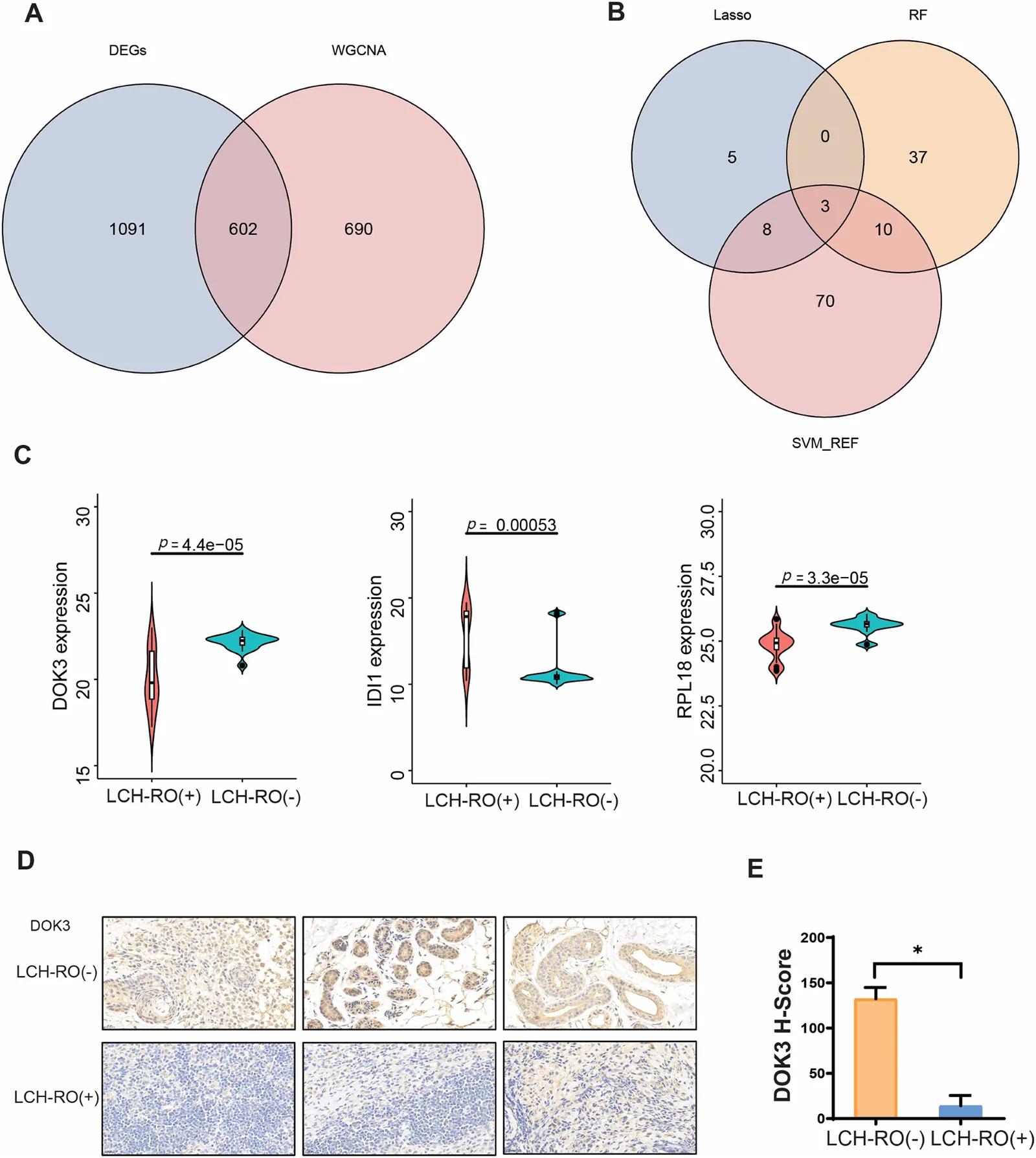
Screening and validation of protein biomarkers for LCH risk stratification. **(A)** Candidate feature proteins were selected by intersecting DEGs with key module proteins from WGCNA. **(B)** Feature selection was performed using LASSO regression, random forest, and SVM-RFE. A Venn diagram shows the three core protein biomarkers commonly identified by all three methods. **(C)** Violin plots display the expression levels of the three core proteins DOK3, RPL18 and IDI1 in LCH-RO (+) and LCH-RO (−) samples. **(D)** Immunohistochemical staining images of DOK3 in MS-LCH and SS-LCH tissues. **(E)** H-Score semi-quantitative analysis of DOK3 expression. *P* < 0.05 indicates statistical significance. * P < 0.05, ** P< 0.01, and *** P< 0.001.

Expression analysis of these three candidate protein biomarkers revealed that DOK3 and RPL18 were downregulated in LCH-RO (+) and upregulated in LCH-RO (–) (**Figure 4C**), whereas IDI1 showed the opposite trend (**Figure 4C**). To further validate these findings, DOK3 protein expression was assessed via immunohistochemistry in three randomly selected patient samples from each of the LCH-RO (+) and LCH-RO (–) groups. The results demonstrated low DOK3 expression in LCH-RO (+) samples and high expression in LCH-RO (–) samples (**Figure 4D**), with H-Score semi-quantitative analysis supporting this pattern (**Figure 4E**). Together, the DOK3 immunohistochemistry findings support the proteomic result, while DOK3, RPL18, and IDI1 remain candidate biomarkers requiring independent validation for distinguishing LCH-RO (−) from LCH-RO (+).

### 3.5 Differences in immune cell infiltration across LCH subtypes

A significant pathological feature of LCH is the infiltration of inflammatory immune cells. Abnormal proportions of various immune cell subsets, including myeloid cells, T cells, dendritic cells, macrophages, and cytotoxic lymphocytes, are commonly observed. The immune microenvironment shaped by these cells is closely associated with the clinical course and severity of LCH, offering important prognostic implications (15, 23, 24). In this study, we compared the infiltration levels of 22 immune cell subtypes between MS-LCH and SS-LCH patient samples. The results revealed significant differences in cellular composition between the two groups (**Figure 5A**). Specifically, MS-LCH samples showed significantly higher infiltration of immune cell types including activated dendritic cells, activated NK cells, resting memory CD4^+^ T cells, CD8^+^ T cells, naive CD4^+^ T cells, and naive B cells. In contrast, significantly lower infiltration was observed for plasma cells, regulatory T cells (Tregs), activated mast cells, eosinophils, resting mast cells, and follicular helper T cells (**Figure 5A**). This infiltration profile suggests that multisystem LCH exhibits a more active innate immune response, accompanied by a relative reduction in immunosuppressive cell populations as well as diminished humoral immunity and certain regulatory/helper T cell components.

**Figure 5:**
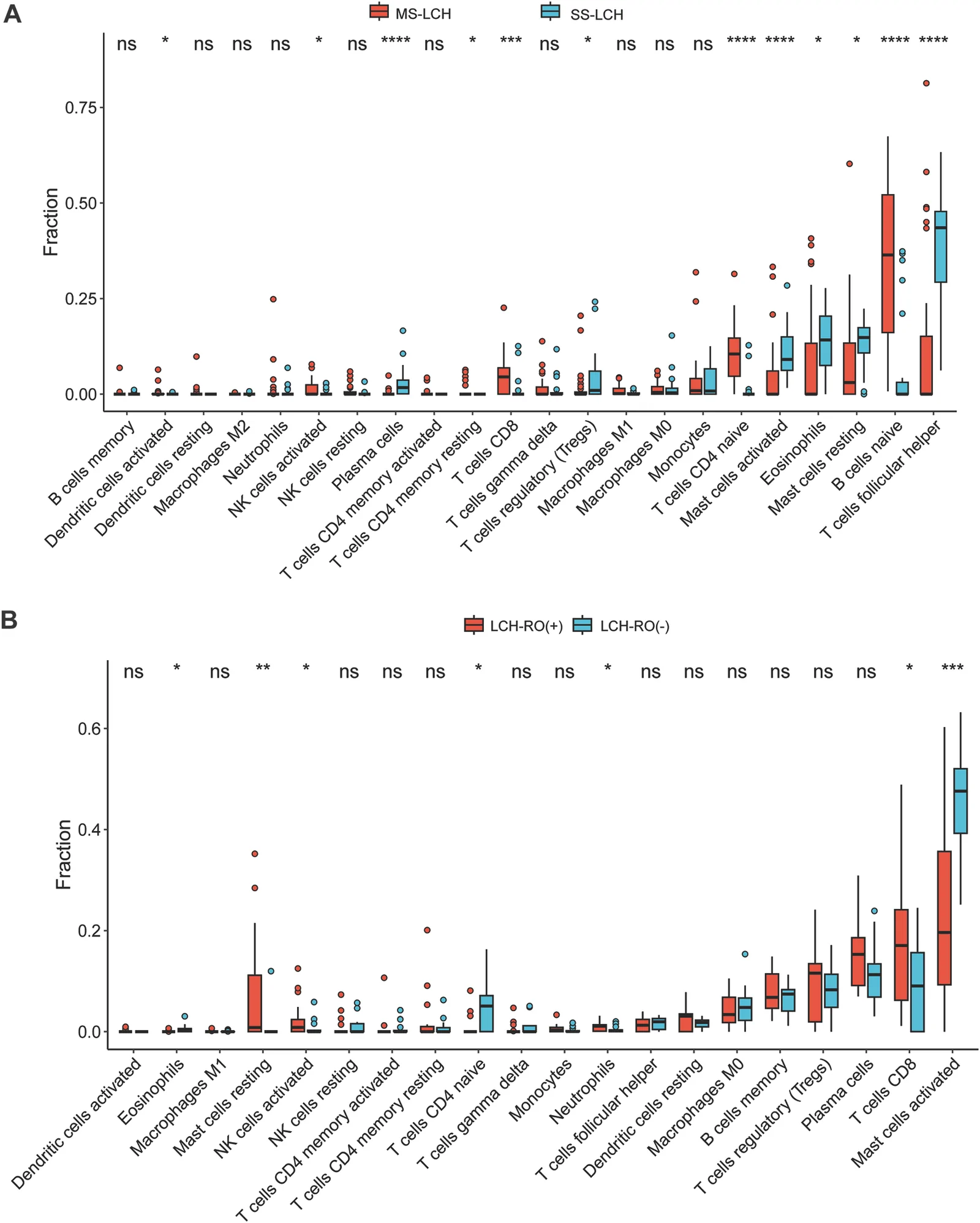
Integrated immune cell infiltration landscape and core protein biomarker correlations in LCH: stratification by disease extent and clinical risk. **(A)** Box plots comparing immune cell infiltration levels between MS-LCH and SS-LCH groups. **(B)** Box plots comparing immune cell infiltration levels between LCH-RO (+) and LCH-RO (−) groups. * P < 0.05, ** P< 0.01, and *** P < 0.001.

In correlation analysis, we found that core protein biomarkers in multisystem LCH showed clear and directionally consistent associations with immune cell infiltration (**Supplementary Figures S6A–G**). For example, BICDL2 was significantly positively correlated with resting mast cells and negatively correlated with macrophages M0 (**Supplementary Figure S6A**); CD22 showed significant positive correlation with naïve B cells and negative correlation with plasma cells (**Supplementary Figure S6B**); CLDN4 was positively correlated with follicular helper T cells but negatively correlated with memory B cells, resting dendritic cells, and regulatory T cells (Tregs) (**Supplementary Figure S6C**); DSC3 was positively correlated with follicular helper T cells and negatively correlated with resting dendritic cells and regulatory T cells (Tregs) (**Supplementary Figure S6D**); ELP1 was significantly positively correlated with resting dendritic cells (**Supplementary Figure S6E**); NAGLU showed no significant correlation with any of the 22 immune cell types (**Supplementary Figure S6F**); and NOSIP was negatively correlated with resting mast cells (**Supplementary Figure S6G**). These findings suggest that key genes in MS-LCH are positively correlated with activated dendritic cells and Tfh cells, and negatively correlated with immunosuppressive or undifferentiated myeloid cells, implying a potential association with immune microenvironment remodeling that warrants further investigation.

Compared to LCH-RO (–), LCH-RO (+) samples exhibited significantly higher infiltration of resting mast cells, activated NK cells, neutrophils, and CD8^+^ T cells, while eosinophils, naive CD4^+^ T cells, and activated mast cells were significantly lower in the LCH-RO (+) group (**Figure 5B**). Correlation analysis between protein biomarkers and immune cell infiltration in the LCH-RO (+) group further supported these immune compositional differences, demonstrating consistency between molecular-level changes and cellular-level immune shifts (**Supplementary Figures S6A–C**). For instance, DOK3 showed significant positive correlation with activated memory CD4^+^ T cells but negative correlation with follicular helper T cells and neutrophils (**Supplementary Figure S7A**); IDI1 was positively correlated with activated dendritic cells (**Supplementary Figure S7B**); and RPL18 was positively correlated with resting dendritic cells (**Supplementary Figure S7C**). Together, these results indicate that LCH-RO (+) patients exhibit prominent features of innate immune activation and enhanced cytotoxic T cell responses, alongside significant reductions in adaptive immune components such as eosinophils, naive/helper T cells, and activated mast cells. The correlation patterns between key proteins such as DOK3, IDI1, and RPL18 and various immune cell subsets further support an overall shift in the immune microenvironment of LCH-RO (+) toward an inflammatory and effector-oriented profile.

### 3.6 Exploratory internal classification analysis based on candidate protein biomarkers

Using the previously identified core protein biomarkers, we developed machine-learning models for two classification tasks: distinguishing multisystem LCH from single-system LCH and identifying patients with risk-organ involvement. Seven commonly used algorithms were systematically compared, including logistic regression, support vector machine (SVM), random forest (RF), XGBoost, naïve Bayes (NB), k-nearest neighbors (KNN), and multilayer perceptron (MLP). Model performance was evaluated using accuracy, the area under the receiver operating characteristic curve (AUC), and the F1 score, together with their corresponding 95% confidence intervals.

For the classification of multisystem versus single-system LCH, most algorithms demonstrated high classification performance in both the training and internal evaluation set (**Figure 6A**). Considering the overall performance metrics and the consistency between the training and validation sets, the SVM model was selected as the representative model. In the internal evaluation set, the SVM model achieved an accuracy, AUC, and F1 score of 1.000, with corresponding 95% confidence intervals of 1.000–1.000. The validation ROC curve showed an AUC of 1.000 (95% CI: 1.000-1.000) (**Figure 6C**). The model-derived scores showed complete separation between multisystem and single-system LCH in this dataset (**Figure 6E**).

**Figure 6:**
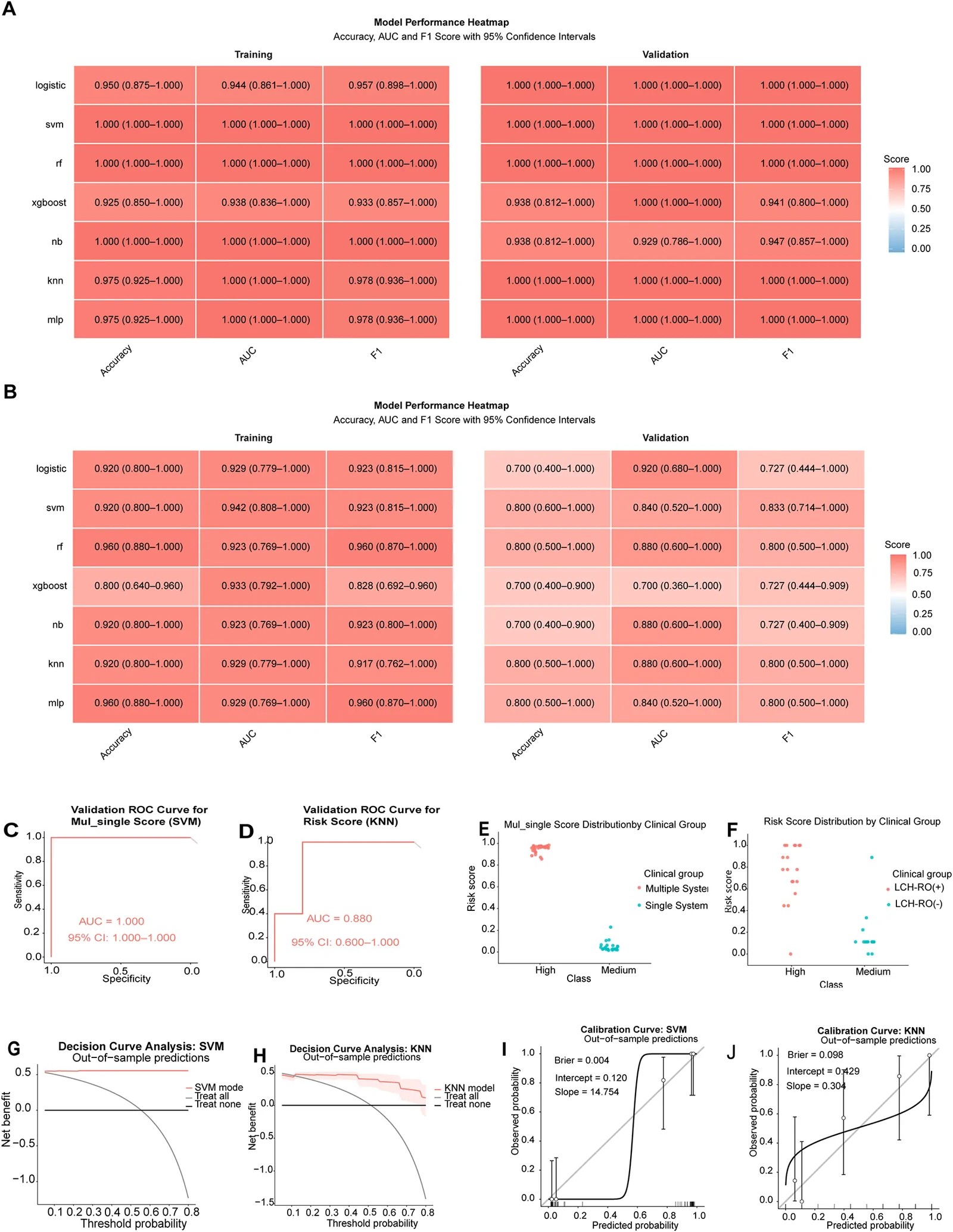
Machine learning–based classification models for distinguishing LCH disease extent and risk stratification. **(A, B)** Performance heatmaps showing accuracy, area under the receiver operating characteristic curve (AUC), and F1 score with 95% confidence intervals for seven classifiers in the training and internal evaluation set. SVM and KNN were selected as representative models for disease extent classification and risk stratification, respectively. **(C, D)** Validation ROC curves for SVM (AUC = 1.000, 95% CI: 1.000–1.000) and KNN (AUC = 0.880, 95% CI: 0.600–1.000). **(E, F)** Score distributions showing clear separation between single-system and multisystem LCH for SVM and partial separation between LCH-RO (−) and LCH-RO (+) for KNN. **(G, H)** Decision curve analyses based on out-of-sample predictions, showing potential net benefit of the SVM and KNN models over the treat-all and treat-none strategies across a range of threshold probabilities. **(I, J)** Calibration curves based on out-of-sample predictions. The SVM model showed a Brier score of 0.004, calibration intercept of 0.120, and slope of 14.754, whereas the KNN model showed a Brier score of 0.098, calibration intercept of 0.429, and slope of 0.304.

For the classification of risk-organ involvement, model performance varied across algorithms (**Figure 6B**). Based on the combined assessment of accuracy, AUC, F1 score, and the consistency of performance between the training and validation sets, the KNN model was selected as the representative model. In the internal evaluation set, the KNN model achieved an accuracy of 0.800 (95% CI: 0.500-1.000), an AUC of 0.880 (95% CI: 0.600-1.000), and an F1 score of 0.800 (95% CI: 0.500-1.000). The validation ROC curve yielded an AUC of 0.880 (95% CI: 0.600-1.000) (**Figure 6D**). The model-derived scores showed partial separation between the LCH-RO (+) and LCH-RO (−) groups (**Figure 6F**). Decision curve analysis showed that, across the evaluated threshold-probability ranges, the net benefits of the SVM and KNN models were generally higher than those of the treat-all and treat-none strategies (**Figures 6G, H**). Calibration analysis showed that the SVM model had a Brier score of 0.004, a calibration intercept of 0.120, and a calibration slope of 14.754 (**Figure 6I**). The corresponding values for the KNN model were 0.098, 0.429, and 0.304, respectively (**Figure 6J**).

## 4 Discussion

This study systematically delineates the proteomic landscape of childhood LCH for the first time. As a rare disease, prior research on LCH has largely been confined to transcriptomic and single-cell analyses, leaving a gap in proteomic-level investigations (25, 26). By integrating FFPE tissue samples from 56 pediatric LCH patients collected over four years at our center, we identified a total of 7207 proteins using mass spectrometry, demonstrating considerable coverage depth and data reliability. Drawing on the successful application of FFPE-based proteomics in biomarker discovery and subtyping across multiple solid tumors (27), we identified several significantly activated functional modules across overall LCH, MS-LCH, and risk-organ-involved [LCH-RO (+)] subtypes, thereby filling a critical research gap at the functional proteomic level in this disease (28). These findings offer novel insights into the clinical heterogeneity, biological mechanisms, and therapeutic strategies of LCH.

Unlike SS-LCH, which involves only a single organ, MS-LCH can affect multiple sites including the skin, bones, lungs, liver, spleen, bone marrow, and central nervous system. Its diagnosis often requires pathological biopsy of involved tissues from multiple sites and evaluation by experienced pathologists (17). In this study, we employed a proteomic approach to systematically explore protein markers that may be used for the clinical subtyping of Langerhans cell histiocytosis (LCH).

In the present study, PCA and PERMANOVA demonstrated that anatomical origin contributed significantly to overall proteomic heterogeneity, with biopsy site accounting for approximately 11.4% of the total proteomic variation. This finding indicates that tissue origin represents a genuine source of variation in bulk LCH proteomes and should not be ignored when comparing heterogeneous LCH lesions with normal skin controls. Nevertheless, the tissue-matched sensitivity analysis restricted to skin-derived LCH lesions showed strong concordance with the primary analysis (Spearman ρ = 0.882), indicating that the major disease-associated effect sizes were largely retained after controlling for tissue origin by restriction. Biopsy-site heterogeneity may also represent a potential source of confounding in proteomic comparisons across LCH clinical subtypes, as different anatomical sites have distinct tissue compositions and protein-expression profiles. However, stratified sensitivity analyses showed that, in both skeleton-derived and skin-derived samples, the protein-level effect sizes for the MS-LCH versus SS-LCH and LCH-RO (+) versus LCH-RO (−) comparisons remained significantly correlated with those obtained from the full cohort. Moreover, the major candidate proteins retained both the same direction of change and statistical significance after stratification by biopsy site. These findings suggest that, although anatomical origin contributes to part of the observed proteomic heterogeneity, it does not fully account for the subtype-associated proteomic differences identified between MS-LCH and SS-LCH or between LCH-RO (+) and LCH-RO (−). Taken together, anatomical origin appears to contribute to, but does not fully account for, the observed proteomic differences. Because matched normal control tissues from skeletal and other involved sites were unavailable, and because some biopsy-site-stratified subgroups remained relatively small and unevenly distributed, residual tissue-of-origin confounding cannot be completely excluded. Accordingly, disease-versus-control findings, particularly those involving tissue-specific biological processes, should be interpreted cautiously, and the subtype-associated findings warrant further validation in larger independent cohorts with a more balanced distribution of anatomical biopsy sites.

By comparing the proteomic profiles of MS-LCH and SS-LCH samples and integrating machine-learning approaches, we identified BICDL2, CD22, CLDN4, DSC3, ELP1, and NOSIP as candidate proteins associated with disease extent. The expression patterns of selected candidates were further supported by immunohistochemistry. These findings provide candidate molecular markers for further investigation of LCH clinical subtypes; however, their classification and clinical utility require validation in independent cohorts. Regarding risk-organ involvement, DOK3, RPL18, and IDI1 showed significant expression differences between LCH-RO (+) and LCH-RO (−), suggesting their potential association with clinical risk status. These proteins therefore represent candidate biomarkers for further investigation rather than established markers for clinical risk stratification.

LCH lesions are highly heterogeneous in composition. Besides LCH cells, they also consist of various immune cells such as T cells, myeloid-derived suppressor cells, macrophages, plasmacytoid dendritic cells, eosinophils, B cells, and multinucleated giant cells. The continuous and mutual interactions between LCH cells and infiltrating immune cells establish a highly inflammatory microenvironment that plays a crucial role in the onset and progression of LCH (15, 16, 29). In this study, we have for the first time delineated the differences in immune cell infiltration between SS-LCH and MS-LCH samples, as well as between LCH-RO (−) and LCH-RO (+) samples. Our findings reveal that MS-LCH patients exhibit a state of systemic immune cell mobilization (e.g., activated dendritic cells, activated NK cells, naive T and B cells) coupled with dysfunction in key effector pathways, such as significant downregulation of plasma cells, regulatory T cells (Tregs), and follicular helper T cells. Compared to LCH-RO (−) patients, those with LCH-RO (+) further display enhanced inflammatory/cytotoxic features characterized by elevated levels of resting mast cells, activated NK cells, neutrophils, and CD8^+^ T cells, alongside further depletion of immune subsets including eosinophils, naive CD4^+^ T cells, and activated mast cells. By integrating the core protein biomarkers identified through machine learning with immune cell infiltration analyses, we further uncovered significant correlations between these core protein biomarkers and specific immune cell subsets.

The machine-learning analyses provided exploratory evidence that the identified protein features may contain information related to LCH disease extent and risk-organ involvement. SVM showed complete separation between multisystem and single-system LCH within the current dataset, whereas KNN showed partial discrimination for risk-organ involvement. However, these results should not be interpreted as validated predictive performance. Although cross-validation and nested resampling were performed, candidate features were selected using the complete discovery cohort before subsequent model evaluation, information from samples later included in the internal evaluation folds contributed to feature selection, resulting in potential information leakage and optimistic performance estimates (30). This concern is particularly relevant to the apparently perfect classification observed for the SVM model. In addition, the markedly abnormal calibration slope of the SVM model and the wide confidence intervals of the risk-organ model further indicate instability related to the limited sample size. Therefore, the present machine-learning results should be regarded strictly as exploratory internal classification findings rather than validation of clinically applicable predictive models. Although decision curve analysis suggested potential net benefit over treat-all or treat-none strategies across some threshold probabilities, these findings are also exploratory and should not be interpreted as evidence of established clinical utility. Prospective evaluation using a completely independent external cohort and a leakage-free modeling pipeline is required before any clinical application can be considered. Regarding the statistical analysis, the lack of FDR correction may increase the false-positive rate. Given the substantial within and between group variability in protein abundance and the limited sample size, we applied a combined differential-selection criterion incorporating both a nominal *P*-value threshold and a fold-change threshold.

Tissue heterogeneity and bulk proteomic measurements may have been influenced by variable cell compositions and local microenvironments. Immune infiltration estimates, derived from computational deconvolution, depend on reference signatures and algorithmic assumptions (31), and cannot replace experimental validation. FFPE-induced protein modifications, degradation, and limited coverage of low-abundance immune markers may further affect quantification and inference accuracy (32). Nevertheless, previous studies have applied CIBERSORT/CIBERSORTx-based deconvolution approaches to bulk proteomic datasets, supporting the feasibility of this strategy for exploratory inference of immune-cell composition (33, 34). Moreover, discordance between mRNA and protein levels (35) and activation-state-associated expression changes may also influence results. Accordingly, immune infiltration findings should be interpreted as exploratory relative trends rather than absolute proportions. Future multicenter prospective cohorts, external validation, and tissue and cell level experimental studies are needed to confirm these findings.

## Data Availability

The datasets presented in this study can be found in online repositories. The names of the repository/repositories and accession number(s) can be found in the article/**Supplementary Material**.
