# Supplementary material for: Integrating proteomics to map immune landscape and protein biomarkers for risk stratification in childhood Langerhans cell histiocytosis
Source: Front Immunol. 2026 Oct 21;17:1909960. doi: 10.3389/fimmu.2026.1909960 (PMC13731401; doi:10.3389/fimmu.2026.1909960)
Supplement: Supplementary file 1 [file Table4.docx]

**Supplementary Information for manuscript “Integrating proteomics to map immune landscape and protein biomarkers for risk stratification in childhood Langerhans cell histiocytosis”**

**Supplementary Figure 1 Influence of biopsy site on LCH proteomic profiles.** (A) PCA and PERMANOVA analyses of LCH samples stratified by biopsy site (R² = 0.114, F = 3.416, *P* = 0.001). (B) Correlation of protein-level effect sizes between the primary analysis and the skin-restricted sensitivity analysis (Spearman ρ = 0.882, *P* < 2 × 10⁻⁶).

**Supplementary Figure 2 Concordance between site-specific and overall proteomic effects.** (A-D) Scatter plots showing correlations between protein-level log2 fold changes from biopsy-site-stratified analyses and those from the overall cohort for MS-LCH versus SS-LCH (A-B) and LCH-RO (+) versus LCH-RO (−) (C-D) comparisons. Significant positive correlations were observed in both skeleton- and skin-derived samples, supporting the overall consistency of subtype-associated proteomic changes across biopsy sites.

**Supplementary Figure 3 Differential protein expression and functional enrichment analysis across LCH subtypes. (A)** Volcano plot showing differentially expressed proteins between MS-LCH and SS-LCH, between LCH-RO (+) and LCH-RO (−) **(C)**. In both plots, red points indicate significantly upregulated proteins, and blue points indicate significantly downregulated proteins. Significance thresholds: upregulated (*P* < 0.05 and log₂FC > 0.58, i.e., FC > 1.5), downregulated (*P* < 0.05 and log₂FC < −0.58), nonsignificant otherwise. **(B)** Bubble plot displaying GO enrichment results for differentially expressed proteins between MS-LCH and SS-LCH. **(D)** Bubble plot displaying GO enrichment results for differentially expressed proteins between LCH-RO (+) and LCH-RO (−). Red dots represent pathways enriched for upregulated proteins (*P* < 0.05), and blue dots represent pathways enriched for downregulated proteins (*P* < 0.05).

**Supplementary Figure 4 Weighted correlation network analysis (WGCNA) of LCH proteomics data. (A)** Left: Scale independence across soft-thresholding powers. Right: Mean connectivity across soft-thresholding powers. Based on the left plot, a soft-thresholding power of 4 was selected to construct a scale-free co-expression network using proteomic data from LCH and control samples. **(B)** Heatmap showing correlations between co-expression modules and LCH. **(C)** Parameter selection for network construction in the comparison between MS-LCH and SS-LCH. Left: Scale independence analysis. Right: Mean connectivity analysis. **(D)** Heatmap showing correlations between co-expression modules and multisystem-LCH. **(E)** Parameter selection for network construction in the comparison between LCH-RO (+) and LCH-RO (−). Left: Scale independence analysis. Right: Mean connectivity analysis. **(F)** Heatmap showing correlations between co-expression modules and LCH-RO (+) versus LCH-RO (−).

**Supplementary Figure 5 Machine learning feature selection for different LCH classifications. (A–B)** Cross-validation error curve and coefficient path plot for LASSO regression in MS-LCH versus SS-LCH. **(C-D)** Five-fold cross-validation error and accuracy curves for SVM-RFE in MS-LCH versus SS-LCH, used to evaluate the classification performance of optimal feature sets. **(E)** Importance ranking of the top 50 feature proteins in the random forest model for MS-LCH versus SS-LCH. **(F–G)** Cross-validation error curve and coefficient path plot for LASSO regression in LCH-RO (+) versus LCH-RO (−). **(H–I)** Five-fold cross-validation error and accuracy curves for SVM-RFE in LCH-RO (+) versus LCH-RO (−), used to evaluate the classification performance of selected features. **(J)** Importance ranking of the top 50 feature proteins identified by the random forest model in the comparison between LCH-RO (+) and LCH-RO (−).

**Supplementary Figure 6 Immune cell infiltration characteristics and their association with core protein biomarkers in multisystem-LCH versus single-system-LCH. (A–G)** Lollipop plots showing correlations between core protein biomarkers (BICDL2, CD22, CLDN4, DSC3, ELP1, and NOSIP) and immune cell infiltration levels in multisystem-LCH samples.

**Supplementary Figure 7 Immune cell infiltration characteristics and their association with core protein biomarkers in LCH risk stratification. (A–C)** Lollipop plots showing correlations between core protein biomarkers (DOK3, RPL18, and IDI1) and immune cell infiltration levels in LCH-RO (+) samples.
